# PACAP and VIP Inhibit the Invasiveness of Glioblastoma Cells Exposed to Hypoxia through the Regulation of HIFs and EGFR Expression

**DOI:** 10.3389/fphar.2016.00139

**Published:** 2016-05-31

**Authors:** Grazia Maugeri, Agata Grazia D’Amico, Rita Reitano, Gaetano Magro, Sebastiano Cavallaro, Salvatore Salomone, Velia D’Agata

**Affiliations:** ^1^Sections of Human Anatomy and Histology, Department of Biomedical and Biotechnological Sciences, University of Catania Catania, Italy; ^2^San Raffaele Open University of Rome Rome, Italy; ^3^Section of Anatomic Pathology, Department of Medical and Surgical Sciences and Advanced Technologies, G.F. Ingrassia, Azienda Ospedaliero-Universitaria “Policlinico-Vittorio Emanuele”, University of Catania Catania, Italy; ^4^Institute of Neurological Sciences, National Research Council Catania, Italy; ^5^Section of Pharmacology, Department of Biomedical and Biotechnological Sciences, University of Catania Catania, Italy

**Keywords:** PACAP, VIP, glioblastoma multiforme invasiveness, hypoxia, HIFs, EGFR

## Abstract

Pituitary adenylate cyclase-activating polypeptide (PACAP) and vasoactive intestinal peptide (VIP) through the binding of vasoactive intestinal peptide receptors (VIPRs), perform a wide variety of effects in human cancers, including glioblastoma multiforme (GBM). This tumor is characterized by extensive areas of hypoxia, which triggers the expression of hypoxia-inducible factors (HIFs). HIFs not only mediate angiogenesis but also tumor cell migration and invasion. Furthermore, HIFs activation is linked to epidermal growth factor receptor (EGFR) overexpression. Previous studies have shown that VIP interferes with the invasive nature of gliomas by regulating cell migration. However, the role of VIP family members in GBM infiltration under low oxygen tension has not been clarified yet. Therefore, in the present study we have investigated, for the first time, the molecular mechanisms involved in the anti-invasive effect of PACAP or VIP in U87MG glioblastoma cells exposed to hypoxia induced by treatment with desferrioxamine (DFX). The results suggest that either PACAP or VIP exert an anti-infiltrative effect under low oxygen tension by modulating HIFs and EGFR expression, key elements involved in cell migration and angiogenesis. These peptides act through the inhibition of PI3K/Akt and MAPK/ERK signaling pathways, which are known to have a crucial role in HIFs regulation.

## Introduction

Pituitary adenylate cyclase-activating polypeptide (PACAP) and vasoactive intestinal peptide (VIP) belong to a family of polypeptides, comprising also peptide histidine-methionine (PHM), secretin, glucagon, glucagon-like peptide (GLP), glucose-dependent insulinotropic polypeptide (GIP), growth hormone releasing hormone (GHRH), and helodermin (Sherwood et al., 2000). They have sequence homology and explicate their functions by binding to vasoactive intestinal peptide receptors (VIPRs) including PAC1, VPAC1, and VPAC2 receptors.

Pituitary adenylate cyclase-activating polypeptide interacts, with high affinity, to PAC1 receptor, whereas both PACAP and VIP can activate either VPAC1 or VPAC2 receptor (Harmar et al., 2012; Tang et al., 2014). VIPRs consist of seven transmembrane domains, a large N-terminal fragment that includes the binding site for PACAP/VIP and an intracellular C-terminal region coupled to heterodimeric G-proteins associated with various signal transduction pathways (Laburthe et al., 2002). Indeed, their stimulation ultimately results in the activation of Protein Kinase A (PKA) (Couvineau et al., 2003; Dickson et al., 2006; Dickson and Finlayson, 2009; Vaudry et al., 2009), Protein Kinase C (PKC) (Vaudry et al., 2000), MAPKs (mitogen-activated protein kinases) (Barrie et al., 1997; Lelièvre et al., 1998), NF-κB signaling pathways (Delgado and Ganea, 1999).

Pituitary adenylate cyclase-activating polypeptide and VIP perform a regulatory role as neurotransmitters and/or neuromodulators in the peripheral and central nervous system (CNS). They are involved in different biological processes such as neuronal survival, stress response, cell division, neuro-, and glio-protective actions (Cavallaro et al., 1995; Canonico et al., 1996; D’Agata et al., 1996; D’Agata and Cavallaro, 1998; Jaworski, 2000; Castorina et al., 2008, 2010a, 2014; Giunta et al., 2012). Furthermore, they play neuroprotective action in various neurodegenerative diseases, comprising Parkinson’s disease (Brenneman, 2007; Dejda et al., 2008; Reglodi et al., 2011; Seaborn et al., 2011; Waschek, 2013). Different studies have also shown that these peptides, through the binding to VIPRs, play a wide variety of effects in human tumors, including GBM (Robberecht et al., 1993, 1994; Oka et al., 1998; Reubi et al., 2000; Juarranz et al., 2001; Isobe et al., 2003). However, their role seems to be controversial depending on histopathological features of cancer. In fact, in some instances, PACAP and VIP stimulate tumor mass growth whereas in others they show antiproliferative effect (Castorina et al., 2008, 2012; Giunta et al., 2010; D’Amico et al., 2013).

More recently, Cochaud et al. (2015) have suggested that VIP interferes with the infiltrative nature of GBM by regulating cell migration. This tumor is the most common and malignant form of primary brain cancer in adults (Wen and Kesari, 2008). Its poor prognosis is related to the therapeutic failure, mainly due to its highly invasive features leading to local or distant recurrences. Indeed, neoplastic cells can migrate to the surrounding tissue, travel along white matter tracts and blood vessel walls to reach other brain areas (Nakada et al., 2007; Dunn et al., 2012).

Glioblastoma multiforme, like other solid tumors, contains extensive areas of hypoxia associated to tissue necrosis and development of aberrant neovascularization (Kaur et al., 2005). In fact, in these regions, low oxygen tension induces expression of hypoxia-inducible factors (HIFs) which mediate the adaptive response through new blood vessels formation. These factors are heterodimeric complexes, including an oxygen-labile α- and a more stable β-subunit (ARNT), involved either in physiological or pathological angiogenesis (Semenza, 1999a,b; Maynard and Ohh, 2004).

In humans there are three HIF-α genes: HIF-1α, HIF-2α, and HIF-3α. In particular, HIF-1α is the key modulator in cellular response to low oxygen tension. It is overexpressed in GBM thereby contributing to intense angiogenesis (Kaur et al., 2005; Zagzag et al., 2006). HIF-2α is also induced by hypoxia, but it plays a major role under chronic insult (Tian et al., 1997; Koh et al., 2011). Several studies have associated HIF-2α up-regulation to development of an aggressive tumor phenotype (Helczynska et al., 2008; Qing and Simon, 2009; Koh and Powis, 2012). Moreover, overexpression of both HIF-1α and HIF-2α has been related to poor prognosis of different cancer, including GBM (Holmquist-Mengelbier et al., 2006; Franovic et al., 2009).

In this tumor, these factors are not only involved in angiogenesis but they also stimulate tumor cell migration and invasion (Zagzag et al., 2006; Fujiwara et al., 2007). Furthermore, the hypoxic microenvironment and activation of HIF-2α in the core of solid tumors induces overexpression of epidermal growth factor receptor (EGFR). Amplification and/or mutations of EGFR are commonly found in GMB. EGFR overexpression, indeed, has been recognized as a prognostic marker of advanced tumoral stage, resistance to standard therapy and reduced patient’s survival (Blehm et al., 2006; Halatsch et al., 2006; Franovic et al., 2007). Under low oxygen tension, accumulation of elevated EGFR levels, in turn mediated by increased HIF-2α, participates to autonomy in tumor cell growth through an autocrine signaling mechanism (Franovic et al., 2007).

A previous study has suggested the involvment of VIP in tumor invasion, but the role of PACAP and its receptors in this biological context still needs to be elucidated. Considering the relevance of the hypoxic microenvironment in determining tumor aggressivity, in the present study we investigated the effect of these peptides in the modulation of HIFs and EGFR expression, both key elements involved in cell migration and angiogenesis. To this end, we also analyzed the underlying molecular pathways by focusing on phosphoinositide three kinase (PI3K)/Akt and mammalian mitogen activated protein kinase/Erk kinase (MAPK/ERK) signaling cascades, since, as previously demonstrated, they interfere with HIF-1α and HIF-2α expression (Mottet et al., 2002; Lim et al., 2004; Park et al., 2011; Zhang et al., 2011).

Our results suggest that these peptides exert an anti-invasive action under hypoxia by modulating HIFs and EGFR expression. This effect is mediated through the inhibition of PI3K/Akt and MAPK/ERK signaling pathways. These data confirm previous findings suggesting that tumor microenvironment may act as an oncogene promoter triggering the autonomous growth of tumor cells. Therefore, the identification of molecules modulating the hypoxic event might give new insights in the therapeutic approach to GBM.

## Materials and Methods

### Human Brain Samples and Cell Lines

The study was performed on glioblastoma frozen sections from a sample provided by Anatomic Pathology of “G.F. Ingrassia” Department, after patient’s signed informed consent. Experiments were also carried on human glioblastoma cell line, U87MG (ATCCC number #HTB-14). Cells were cultured in Dulbecco’s modified Eagle’s medium (DMEM) supplemented with 10% of heat-inactivated fetal bovine serum (FBS), 100 U/ml penicillin and 100-μg/ml streptomycin (Sigma–Aldrich, Steinheim, Germany). They were incubated at 37°C in a humidified atmosphere with 5% CO2 as previously described by Maugeri et al. (2016). Cells grown under hypoxia were exposed for 24 h to 100 μM desferrioxamine mesylate salt (DFX) (Sigma–Aldrich), an hypoxia-mimetic agent, which induces hypoxia via inhibition of the HIF prolyl hydroxylases (Epstein et al., 2001; Hirsilä et al., 2005). As compared to the cell incubation method in hypoxic chamber, this offers the advantage to the experimentator to open the culture plate/dish/flask many times without affecting the hypoxic condition.

### Treatments

Pituitary adenylate cyclase activating polypeptide-38 (PACAP38, 100 nM; cat no. A1439, Sigma–Aldrich), VIP, (100 nM; cat no. V3628, Sigma–Aldrich), PACAP receptor antagonist (PACAP6–38, 10 μM; cat no.3236, Tocris Biosciences, Bristol, UK) and selective VIP receptor antagonist (D-p-Cl-Phe6,Leu17-VIP, 10 μM; cat no. 3054, Tocris Biosciences) were added to U87MG cells for 24 h in normoxic or hypoxic condition.

Inhibition of PI3K/Akt and MAPK/ERK signaling pathways was performed following a pretreatment of 30 min with a PI3K (Wortmannin, 10 μM; cat no.1232/5, Tocris Biosciences) or MEK1 inhibitor (PD98059, 50 μM; cat no. #P215, Sigma–Aldrich,) under normoxia or hypoxia (Aleppo et al., 1992; Castorina et al., 2010b).

### Cell Migration Assay

After trypsinization, U87MG cells were resuspended, counted and seeded onto a six-well plate at a density of 5 × 10^4^ cells/well. A scratch was made in cell monolayer with a 200-μL pipette tip. Then, to remove the suspended cells, it was washed twice with PBS and incubated in medium containing 100 nM PACAP38 or 100 nM VIP or 10 μM Wortmannin or 50 μM PD98059, either in normoxic or hypoxic condition. The wounded areas were visualized under a microscope for quantification. The distance that the advancing cells had moved into the cell-free (wound) area was measured after 24 h by staining with crystal violet. Migration was calculated as the average number of cells observed in five random high power wounded fields/per well in duplicate wells.

### Western Blot Analysis

Western blot analysis was performed according to the procedures previously described by Magro et al. (2011). Proteins were extracted with buffer containing 20 mM Tris (pH 7.4), 2 mM EDTA, 0.5 mM EGTA; 50 mM mercaptoethanol, 0.32 mM sucrose and a protease inhibitor cocktail (Roche Diagnostics, Monza, Italy) using a Teflon-glass homogenizer and then sonicated twice for 20 s using an ultrasonic probe, followed by centrifugation at 10,000 × *g* for 10 min at 4°C. Protein concentrations were determined by the Quant-iT Protein Assay Kit (Invitrogen,Carlsbad, CA, USA). About 65 μg from fresh frozen section *per* sample, and about 30 μg of protein homogenate were diluted in 2X Laemmli buffer (Invitrogen), heated at 70°C for 10 min and then separated on a Biorad Criterion XT 4–15% Bis-tris gel (Invitrogen) by electrophoresis and then transferred to a nitrocellulose membrane (Invitrogen). Blots were blocked using the Odyssey Blocking Buffer (Li-Cor Biosciences, Nebraska, NE, USA). The transfer was monitored by a prestained protein molecular weight marker [BioRad Laboratories, Segrate (MI), Italy]. Immunoblot analysis was performed by using appropriate antibodies: rabbit anti-PACAP (H-76, cat no. sc-25439, Santa Cruz Biotechnology, Texas City, TX, USA 1:200), mouse anti-VIP (H-6, cat no. sc-25347, Santa Cruz Biotechnology; 1:100), (rabbit anti-PAC1 receptor (H-55, cat no.sc-30018, Santa Cruz Biotechnology; 1:300), rabbit anti-VPAC1 (H-130, cat no. sc-30019, Santa Cruz Biotechnology; 1:200), rabbit anti-VPAC2 (H-50, cat no. sc-30020, Santa Cruz Biotechnology; 1:200), mouse anti- HIF-1alpha (cat.n. NB100-105, Novus Biologicals, Littleton, CO, USA 1:500), rabbit anti- HIF-2alpha (cat.n. NB100-122, Novus Biologicals, 1:500), rabbit anti- EGFR (cat.n. sc-03 Santa Cruz Biotechnology), rabbit anti-phospho Akt (Ser473 residue) (D9E, cat no. #4060, Cell Signaling, Danver, MA, USA; 1:1000), rabbit anti-total Akt (C67E7, cat no. #4691, Cell Signaling; 1:1000), mouse anti-phospho Erk-1/2 (Thr202 and Tyr204 residues) (pT202/pY204.22A, cat no. sc-136521, Santa Cruz Biotechnology; 1:200), mouse anti-total Erk-1/2 (MK1, cat no. sc-135900, Santa Cruz Biotechnology; 1:200), and rabbit anti-β-tubulin (cat n.sc-9104, Santa Cruz Biotechnology; 1:500). The secondary antibody goat anti-rabbit IRDye 800CW (cat #926-32211; Li-Cor Biosciences) and goat antimouse IRDye 680CW (cat #926-68020D, Li-Cor Biosciences) were used at 1:20,000 and 1:30,000, respectively. Blots were scanned with an Odissey Infrared Imaging System (Odyssey). Densitometric analyses of Western blot signals were performed at non-saturating exposures and analyzed using the ImageJ software^1^ (NIH, Bethesda, MD, USA). Values were normalized to β-tubulin, which served as loading control, as previously described by Maugeri et al. (2015).

### Immunohistochemical Analysis

Fresh-frozen sections of a surgically resected tumor included in OCT were cut and fixed in 4% paraformaldehyde for 30 min. Then, they were treated with 3% H_2_O_2_ in methanol for 10 min to inhibit the endogenous peroxidase activity as previously described by D’Agata et al. (2000). To reduce non-specific staining, sections were treated with 1% bovine serum albumin (BSA) in PBS for 1 h, and incubated overnight at 4°C with appropriate antibody. The sections were rinsed in PBS and incubated with diaminobenzidine (DAB) for 5 min. Hematoxylin was used as nuclear counterstain. The stained sections were dehydrated through graded alcohols, cleared in xylene, and covered with neutral balsam.

### Statistical Analysis

Data are represented as mean ± standard error (SEM). One-way analysis of variance (ANOVA) was used to compare differences among groups, and statistical significance was assessed by the Tukey–Kramer *post hoc* test. The level of significance for all statistical tests was set at *p* ≤ 0.05.

## Results

### Expression of PACAP, VIP, VIPRs, HIF-1α, HIF-2α, and EGFR in Human Glioblastoma

**FIGURE 1:**
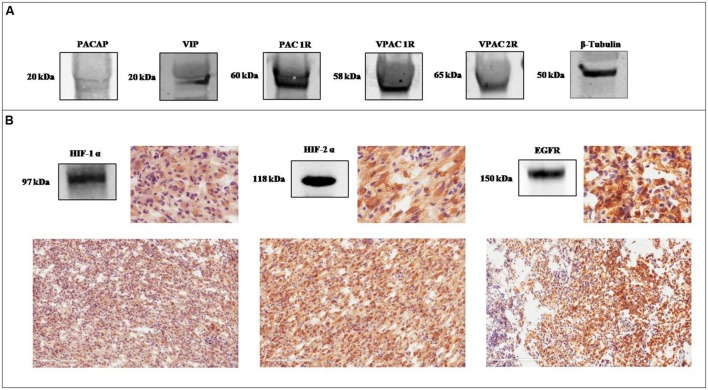
**Expression of PACAP, VIP, VIPRs, HIF-1α, HIF-2α, and EGFR in glioblastoma multiforme (GBM). (A)** Representative immunoblot of PACAP and VIP precursor peptides and PAC1R, VPAC1R, and VPAC2R expression on frozen glioblastoma sample. **(B)** Representative immunoblot and photomicrographs of signals detected by antibodies direct against HIF-1α, HIF-2α, and EGFR in a frozen glioblastoma sample.

As previously demonstrated, here, we found that the precursor proteins of PACAP and VIP, and VIPRs are expressed in GBM; however, precursor peptides levels seemed to be lower as compared to their receptors (**Figure 1A**). To correlate this finding with tumor malignancy, we have confirmed HIF-1α, HIF-2α, and EGFR expression in sections from the same frozen sample by western blot analysis (**Figure 1B**). Furthermore, we have determined their tissue localization by immunohistochemical analysis. An heterogeneous tissue staining of HIF-1α, HIF-2α, and EGFR has been observed in this tumor (**Figure 1B**).

### PACAP and VIP Antagonize Hypoxia-Mediated GBM Cell Migration by Reducing HIFs and EGFR Expression

**FIGURE 2:**
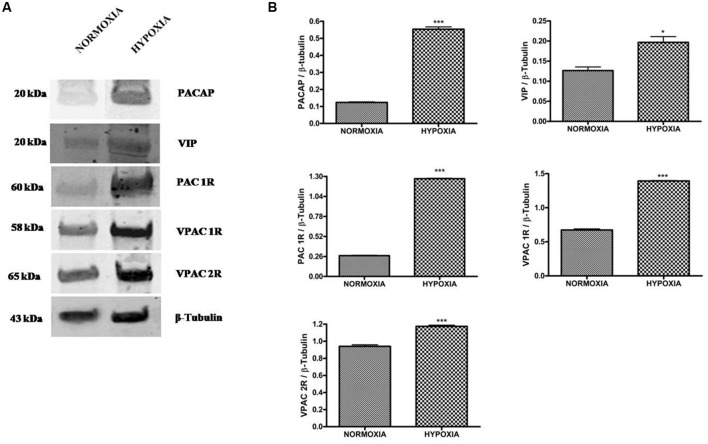
**Expression of PACAP, VIP, PAC1R, VPAC1R, and VPAC2R in glioblastoma cells under normoxic or hypoxic conditions. (A)** Representative immunoblot of PACAP and VIP precursor peptides and PAC1R, VPAC1R, and VPAC2R expression on U87MG cells grown normoxia or exposed to hypoxia. **(B)** The bar graphs show quantitative analysis of signals obtained on immunoblots resulting from three independent experiments. Relative band densities were quantified by using ImageJ software. Protein levels are expressed as arbitrary units obtained after normalization to β-tubulin which was used as loading control. Data represent means ± SEM (*p < 0.05 or ***p < 0.001 vs. Normoxia as determined by unpaired two-tailed Student *t*-test).

**FIGURE 3:**
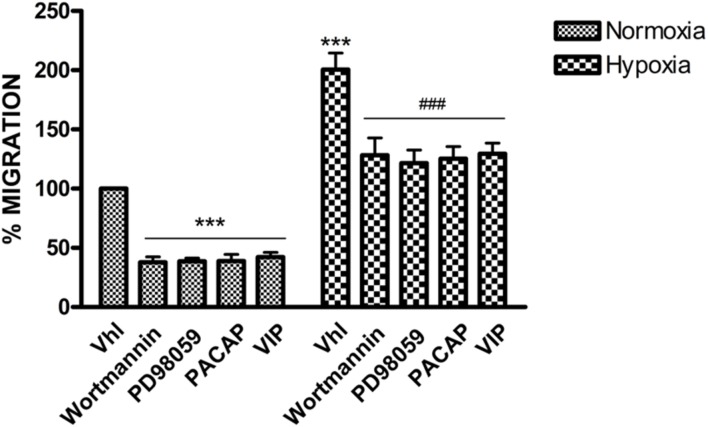
**Effect of PACAP, VIP, PI3K, and MEK1 inhibitors on U87-MG cells migration.** Cells monolayer was scraped by a pipette tip and incubated with PACAP, VIP, Wortmannin or PD98059 for 24 h under normoxia or hypoxia. The wounded areas were visualized under a microscope for quantification. Migration was calculated as the average number of cells observed in five random high power wounded fields/per well in duplicate wells. In the bar graph values are expressed as percentage of control. (***p < 0.001 vs. Vhl under normoxia; **^###^***p* < 0.001 vs. Vhl under hypoxia).

To characterize the role of these peptides in hypoxic areas of GBM, we have analyzed the expression of PACAP or VIP precursor proteins in U87MG tumor cells, grown for 24 h under normoxia or hypoxia. As shown in **Figure 2**, both precursor peptides and VIPRs receptors were expressed in these cells under normal oxygen tension, however, their levels were significantly increased following the hypoxic insult (**Figure 2B**, *p < 0.05 or ***p < 0.001 vs. normoxia). In view of this result, we have investigated the effect of these peptides on proliferation and invasion of gliomas cells during hypoxic process, which represents a distinctive biological feature of malignant cells. Therefore, we have analyzed the effect of 100 nM PACAP and VIP on tumoral cells invasivity. At this concentration, both peptides showed antiproliferative properties, as previously described by D’Amico et al. (2013). The dose used is higher as compared to their tumor tissues level. However, considering that tumoral mass is highly heterogeneous, comprehending various cell types with different mutations and degree of differentiation, in the present study, we decided to omit the characterization of their physiological role in cancer. Then, we have focused on the effect of exogenous peptides in a homogeneous cell culture. As shown in **Figure 3**, migration rate increased in GBM cells exposed to DFX as compared to normoxic control (Vhl). PACAP or VIP treatment significantly decreased cell invasion both under normal or low oxygen tension. Similarly, treatment with Wortmannin or PD98059, a PI3K and MEK1 inhibitors, respectively, induced significant reduction of cell’s migration confirming that tumor cell invasion and proliferation is mediated via activation of PI3K/AKT and ERK pathway (***p < 0.001 vs. Vhl in normoxia; ###*p* < 0.001 vs. Vhl in hypoxia).

**FIGURE 4:**
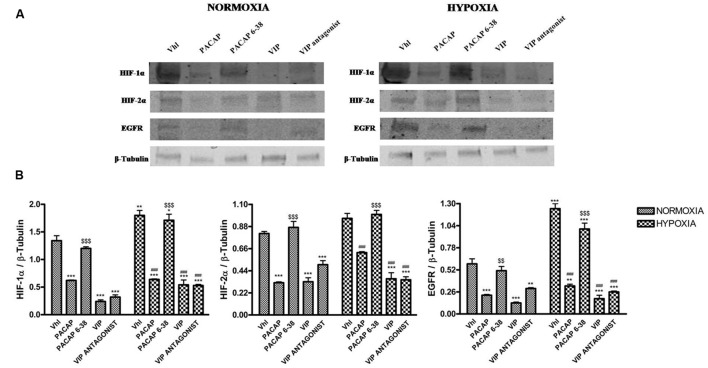
**Effect of PACAP, PACAP6-38, VIP, and VIP antagonist on expression of HIF-1α, HIF-2α, and EGFR in U87-MG cells under normoxia and hypoxia. (A)** Representative immunoblots of HIF-1α, HIF-2α, and EGFR expression on U87MG cells grown normoxia or exposed to hypoxia. **(B)** The bar graphs show quantitative analysis of signals obtained by immunoblots resulting from three independent experiments. Relative band densities were quantified by using ImageJ software. Protein levels are expressed as arbitrary units obtained after normalization to β-tubulin which was used as loading control. Data represent means ± SEM (*p < 0.05, **p < 0.01, or ***p < 0.001 vs. Vhl under normoxia; **^###^***p* < 0.001 vs. Vhl under hypoxia ^$$^*p* < 0.01 or ^$$$^*p* < 0.001 vs. PACAP, as determined by one-way ANOVA followed by the Tukey *post hoc* test).

Based on these results, we further explored whether these peptides have performed their effect through modulation of HIFs and EGFR expression. PACAP treatment induced a significant reduction of HIF-1α, HIF-2α, and EGFR, either in normoxia or hypoxia, as compared to control. Furthermore, to confirm whether the effect of this peptide was mediated through the selective activation of PAC1 receptor, we also treated cells with a PACAP antagonist, such as PACAP 6–38, under normoxic or hypoxic condition. PACAP 6–38 treatment restored the expression of these proteins to their relative control levels, both in normoxia or hypoxia, confirming the involvement of PAC1 receptor (**Figure 4B**, *p < 0.05, **p < 0.01 and ***p < 0.001 vs. Vhl; ^###^*p* < 0.001 vs. Vhl in hypoxia; ^$$^*p* < 0.01 ^$$$^*p* < 0.001 vs. PACAP).

Similarly to PACAP, also VIP treatment significantly reduces expression levels of HIFs and EGFR as compared to relative controls. However, VIP antagonist treatment was not able to restore HIF-1α, HIF-2α, and EGFR expression to control levels (**Figure 4B**, *p < 0.05, **p < 0.01, and ***p < 0.001 vs. Vhl; ^###^*p* < 0.001 vs. Vhl in hypoxia).

### PACAP and VIP Decrease HIFs and EGFR Expression, through Inhibition of PI3K/Akt and MAPK/ERK Pathways

**FIGURE 5:**
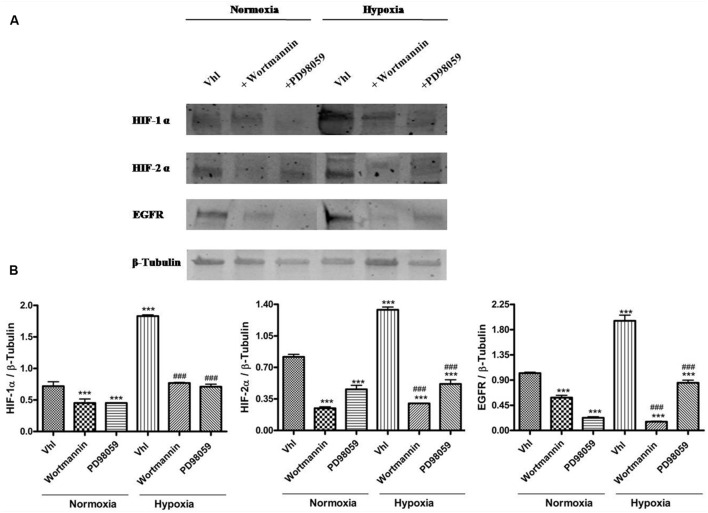
**Expression of HIF-1α, HIF-2α, and EGFR following inhibition of PI3K/Akt or MAPPK/Erk kinase signaling pathway. (A)** Representative immunoblot of HIF-1α, HIF-2α, and EGFR expression on U87MG cells treated with 10 μM Wortmannin or with 50 μM PD98059 and grown normoxia or exposed to hypoxia. **(B)** Relative density of each band was quantified using ImageJ software. Each signal was normalized on correspondent β-tubulin signal. Data are expressed as mean ± SEM (**p < 0.01 and ***p < 0.001 vs. Vhl under normoxia; ^###^*p* < 0.001 vs. Vhl under hypoxia as determined by one-way ANOVA followed by the Tukey *post hoc* test).

Remarkably, PI3K/Akt and the MAPK/ERK signaling cascades are aberrantly activated in many cancers, including GBM. The stimulation of these pathways leads to increase of HIF-1α and HIF-2α levels, which are involved in the aggressive behavior of tumor and promotion of angiogenesis. Furthermore, HIFs promote EGFR activation. To confirm this crucial link, we have treated cells with Wortmannin or PD98059. As shown in **Figure 5**, both substances induced a statistically significant decrease of HIF-1α, HIF-2α and EGFR expression as compared to control cells grown either under normoxia or hypoxia (**Figure 5**, **p < 0.01, ***p < 0.001 vs. Vhl in normoxia; ^###^*p* < 0.001 vs. Vhl in hypoxia).

**FIGURE 6:**
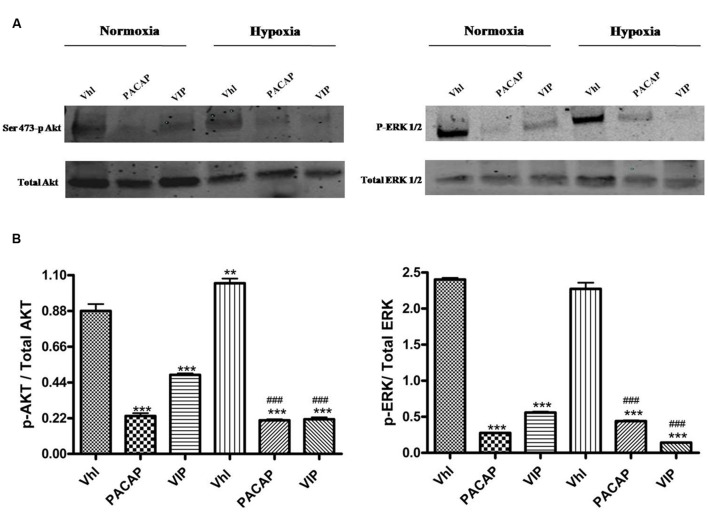
**Phosphorylation of AKT and ERK1/2 in U87-MG cells under normoxia and hypoxia. (A)** Representative immunoblots of Ser473-p Akt or p-Erk1/2 expression on U87MG cells treated with PACAP or VIP under normoxia or hypoxia. **(B)** The bar graphs show quantitative analysis of signals obtained by immunoblots resulting from three independent experiments. Relative band densities were quantified by using ImageJ software. Each signal of phosphorylated protein was normalized to total protein expression. Data are expressed as mean ± SEM (**p < 0.01, ***p < 0.001 vs. Vhl under normoxia; ^###^*p* < 0.001 vs. Vhl under hypoxia as determined by one-way ANOVA followed by the Tukey *post hoc* test).

To investigate whether PACAP and VIP regulate HIFs and EGFR levels, through inhibition of these signaling pathways, we assessed their effect on phosphorylation of two signaling proteins. As shown in **Figure 6**, Akt and ERK1/2 are activated to comparable levels both under normoxia or hypoxia. We have hypothesized that this may be due to lack of the functional gene opposing tumor suppressor lipid phosphatase (PTEN) in the glioma cell line, U87MG, used. This is a tumor suppressor gene acting as a negative regulator of both PI3K/Akt and ERK1/2 signaling pathways (Li et al., 1997; Koul, 2008; Chetram and Hinton, 2012; Song et al., 2012). Instead, the treatment of U87MG cells with these peptides significantly decreased the level of phosphorylated Ser473 AKT and ERK1/2 both in normoxia and hypoxia (**Figures 6A,B**, ***p < 0.001 vs. Vhl in normoxia; ^###^*p* < 0.001 vs. Vhl in hypoxia).

## Discussion

Pituitary adenylate cyclase-activating polypeptide and VIP are widely expressed in peripheral tissues, CNS and in a wide variety of human tumors, including GBM (Robberecht et al., 1993, 1994; Oka et al., 1998; Reubi et al., 2000; Juarranz et al., 2001; Isobe et al., 2003). They exert various effects by interacting to VIPRs depending on transcript variants express in each cell type. By focusing on cancer, many studies have highlighted their controversial role in the progression of malignancies. In particular, they have shown a prominent growth effect on some common neoplasms, such as lung, gut, prostate and immune system neoplastic diseases (Reubi et al., 2000; Moody et al., 2001; Schulz et al., 2004). However, as we show in this paper, they exert anti-invasive effect, in other tumors, including GBM (Vertongen et al., 1996; Cochaud et al., 2015). During tumorigenesis, hypoxic areas are generated in the neoplastic mass when deregulation in cell proliferation leads to an increase in tissue amount, not supported by an adequate oxygen supply. Hypoxia plays a key role in malignancy not only by stimulating angiogenesis but also by increasing cellular migration. Therefore, in the present study, we evaluated the effect of PACAP and VIP on tumor cell infiltration grown in a hypoxic microenvironment. The present results show that the endogenous expression of PACAP and VIP precursor peptides, and relative VIPRs was increased under hypoxia (**Figure 2**). Furthermore, these neuropeptides significantly abrogated the hypoxia-enhanced migration of U87MG cells (**Figure 3**), suggesting that they might play a pivotal role in cellular invasion in GMB hypoxic areas. It is remarkable that HIFs, the main regulators of the transcriptional response to hypoxia, represent one of the distinctive hallmarks in malignancy. Here, we have found that PACAP or VIP treatment, decreases the expression levels of both HIF-1α and HIF-2α. Concomitantly, we have found that their administration have induced a significant reduction in EGFR levels, a biomarker of cell proliferation. The present data, consistently with previous papers, indicate that hypoxia promotes an oncogenic program which results from the translational up-regulation of EGFR, predominantly depending on HIFs levels (Franovic et al., 2007, 2009). This view is also corroborated by the high expression of PACAP, VIP, VIPRs, HIFs, and EGFR in the frozen tumor sample (**Figure 1**).

Furthermore, we have investigated whether modulation of HIFs and EGFR expression is mediated through VIPRs by treating cells with PAC1 and VPAC1/VPAC2 receptor antagonists, respectively. The results show that PACAP 6–38 treatment highly increased HIFs and EGFR levels, suggesting that hypoxia through activation of endogenous PACAP system may interfere with hypoxia and relative cell proliferation mediated by EGFR (**Figure 4**). On the other hand, the VIP receptor antagonist was ineffective (**Figure 4**). This lack of effect might result from a simultaneous block of VPAC1 and VPAC2 receptors, which mediate different functions by activating various pathways, or, more simply, might be due to low specificity of the tested antagonist molecule.

As demonstrated previously, HIFs expression is regulated by PI3K/Akt and MAPK/ERK signaling pathways (Mottet et al., 2002; Lim et al., 2004). In addition, Akt phosphorylation promotes the transformation of anaplastic astrocytoma in GBM, thereby playing a role as oncogenic modulator. In fact, this molecule is involved in cell proliferation by acting on some regulators of cell cycle, apoptosis, and metabolism (Sonoda et al., 2001a,b). MAPK/ERK pathway activation is also involved in tumorigenesis by supporting progression and poor-prognosis of GBM (Kim et al., 2009; Liu et al., 2013).

Our results have confirmed previous data suggesting that the expression of HIFs and consequently EGFR is mediated by activation of both pathways. Indeed, as shown in **Figure 5**, the pretreatment with a specific PI3K/Akt (wortmannin) or MAPK/ERK (PD98059) pathway inhibitor, strongly decrease HIFs and EGFR levels as compared to own control, in cells grown either in normoxia or exposed to DFX-induced hypoxia. In future, we are planning to deeply investigate the downstream phenotypic effects mediated by EGFR under these experimental conditions.

Thus, we have demonstrated that the anti-invasive effect of PACAP and VIP in GBM cells is mediated through inhibition of these pathways. Indeed, the treatment with these peptides reduces Akt and ERK1/2 phosphorylation which are major targets of PI3K/Akt or MAPK/ERK signaling cascades, respectively (**Figure 6**).

Apparently, these results are in contrast with other studies showing that PACAP and VIP, through the activation of cAMP/PKA and PI3K signaling pathways, mediated by VIPRs, stimulate a series of transcription factors, which promote proliferation, expression of nuclear oncogenes and growth factors in different cell lines (Whitmarsh and Davis, 1996; Casibang et al., 2001). However, this controversial biological effect might depend by cell or tissue phenotype examined.

In conclusion, these data suggest that, under low oxygen tension, PACAP or VIP reduce cell invasion by acting as negative regulators of HIFs and EGFR through the inhibition of PI3K/Akt and ERK1/2 signaling pathways. Further studies are required to clarify whether PACAP and VIP have the same effects, as observed in the present study, in GBM cells exposed to hypoxia, not chemically induced, but through lowering of oxygen tension (1% O_2_).

Despite over the last three decades have emerged new treatments of brain tumors, the survival of patients with GBM remains very poor. Therefore, new targeted agents in clinical therapy are needed. Here, we propose that the modulation of PACAP and VIP receptors system in combination with other therapies, might represent a new approach to limit invasion of this devastating tumor.

## Conclusion

The modulation of hypoxic event and the anti-invasive effect exerted by some VIP family members might open new insights in the therapeutic approach to GBM.

## Author Contributions

VD: Study conception and design; Drafting of manuscript; Critical revision. GM: Study conception and design; Acquisition of data; Analysis and interpretation of data; Drafting of manuscript. AGD: Acquisition of data; Analysis and interpretation of data. RR: Acquisition of data; Analysis and interpretation of data. GM: IHC analysis; Critical revision. SC: Critical revision. SS: Drafting of manuscript; Critical revision.

## Conflict of Interest Statement

The authors declare that the research was conducted in the absence of any commercial or financial relationships that could be construed as a potential conflict of interest.

## Footnotes

1 http://rsb.info.nih.gov/ij/index.html
